# A Missing Puzzle in Preclinical Studies—Are CCR2, CCR5, and Their Ligands’ Roles Similar in Obesity-Induced Hypersensitivity and Diabetic Neuropathy?—Evidence from Rodent Models and Clinical Studies

**DOI:** 10.3390/ijms252011323

**Published:** 2024-10-21

**Authors:** Aleksandra Bober, Joanna Mika, Anna Piotrowska

**Affiliations:** Department of Pain Pharmacology, Maj Institute of Pharmacology, Polish Academy of Sciences, 31-343 Krakow, Poland; bober@if-pan.krakow.pl

**Keywords:** neuropathy, diabetes, obesity

## Abstract

Research has shown that obesity is a low-grade inflammatory disease that is often associated with comorbidities, such as diabetes and chronic pain. Recent data have indicated that chemokines may play a role in these conditions due to their pronociceptive and chemotactic properties, which promote hypersensitivity and inflammation. Accumulating evidence suggests that CCR2, CCR5, and their ligands (CCL2, CCL3, CCL4, CCL5, CCL7, CCL8, CCL11 CCL12, and/or CCL13) play a role in rodent models of pain and obesity, as well as in patients with diabetes and obesity. It was proven that the blockade of CCR2 and CCR5, including the simultaneous blockade of both receptors by dual antagonists, effectively reduces hypersensitivity to thermal and mechanical stimuli in chronic pain states, including diabetic neuropathy. The present review discusses these chemokine receptors and the role of their ligands in diabetes and obesity, as well as their involvement in diabetic neuropathy and obesity-induced hypersensitivity.

## 1. Introduction

In 2019, the global prevalence of diabetes was assessed to be 9.3%, and it is estimated to reach 10.2% by 2030 [1]. According to the World Health Organization (WHO), the number of people with diabetes increased from 108 million in 1980 to 422 million in 2014 [2]. In a similar timeframe (1990–2022), adult obesity has more than doubled, and adolescent obesity has quadrupled, resulting in an alarming number of obese individuals, with 1 in 8 people worldwide struggling with obesity [3]. Both of these diseases are interlinked, as obesity is a frequent risk factor for type 2 diabetes mellitus (T2DM), and there is a direct correlation between increased body mass index (BMI) and the risk of developing T2DM; moreover, drugs that are used to treat T2DM, such as semaglutide, have beneficial effects on weight loss in obese patients [4]. High BMI and poor glycemic control are markers associated with painful diabetic neuropathy [5], because hyperglycemia leads to nerve damage [5,6] and can result in serious complications, including diabetic foot syndrome [7], being the first cause of non-traumatic amputations of the lower limb in western countries [8]. Diabetic neuropathy is the most prevalent complication of diabetes and it is estimated to affect up to 50% of patients with diabetes [5,9,10,11]. The prevalence of diabetic neuropathy is similar in type 1 diabetes mellitus (T1DM) and T2DM patients [5], but there is a higher incidence in T2DM patients [5,11]. In addition, women are at increased risk of developing painful symptoms of diabetic neuropathy [5,12,13]; however, often female animals have not been included in preclinical research, and sex differences have not been studied in clinical trials [14]. Drugs that are used to treat diabetic neuropathic pain include anticonvulsants, antidepressants, opioids, topical capsaicin, and topical lidocaine, but their analgesic effects are often insufficient [15,16,17,18]. Anticonvulsants, including selective serotonin and noradrenaline reuptake inhibitors, and tricyclic antidepressants are used as first-line therapies [17]. These drugs often need titration and cause adverse effects, such as somnolence, dizziness, fatigue, and nausea [15,16,17,18]. Opioid use is only recommended in patients who are unresponsive to standard treatment, because opioid use is associated with the risk of substance dependence and abuse, including overdose. Additionally, opioids can induce opioid tolerance or hyperalgesia [15]. Patients with obesity more often experience pain, including chronic pain and unrelieved pain, compared to nonobese individuals [19,20,21,22]. Patients with a high BMI are more likely to develop low back pain, abdominal pain, headaches, tension-type headaches, migraine headaches, pelvic pain, joint pain, fibromyalgia, chronic widespread pain, and neuropathic pain [21,22]. Data from the literature show that the prevalence of neuropathy is greater in obese patients with diabetes than in obese patients with prediabetes and in obese patients with normoglycemia; however, obese patients with normoglycemia are still 2.9–5.5 times more likely to experience neuropathy than lean controls. These data suggest that diabetes is an important factor in the development of neuropathic pain, but obesity alone can also lead to neuropathy [23,24].

Diabetic neuropathy is a result of different pathological mechanisms, including metabolic disorders, microvascular damage, demyelination, and changes in the interactions between neuronal and immunological systems [5,25,26,27,28]. Diabetes often affects the peripheral nervous system, in which neurons’ cell bodies are located outside the blood–brain barrier, making them more vulnerable to damage resulting from diabetes [29]. The reaction to noxious stimuli (like pressure, touch, and cold) is driven by lightly myelinated Aδ and unmyelinated C fibers, while the response to non-noxious stimuli is led by highly myelinated Aβ fibers [29,30]. The initial phase of neurodegeneration consists of demyelination or remyelination of the large fibers, which causes early deterioration and loss of C and Aδ fibers, and then large-fiber axonal loss. Diabetic neuropathy is defined by distal-to-proximal axonal loss, which is a result of mitochondrial dysfunction and the accumulation of toxic substances like acylcarnitine and reactive oxygen species. Insufficient energy and lack of protection from overloaded Schwann cells cause small fibers to degrade first [29,31]. However, the background of diabetic neuropathic pain is complex and it also involves neuroimmune crosstalk. In hyperglycemic conditions, non-neuronal cells, like microglia, astrocytes, and immune cells, are activated in the spinal cord [32,33], which plays an important role in the development of neuropathic pain [34]. Activated cells contribute to neuropathic pain by releasing proinflammatory cytokines, including chemokines [34,35,36,37,38,39]. An increase in the chemokines produced by glial and immune cells can lead to the activation of CCR2 and CCR5 receptors located on spinal cord neurons and their sensitization, which contributes to the initiation of a cascade of events leading to the development of hypersensitivity and is of fundamental importance in the development of neuropathic pain. Some of the chemokines and their receptors are postulated to be biomarkers for diabetes or diabetic neuropathy [40,41]. For example, one of the studies has shown that CCL2 was significantly correlated with standard diabetes-related parameters like glycemic excursion and HbA1c [41]. However, more research is needed in this topic to investigate the possible correlation between diabetic neuropathy status and basic biochemical parameters in diabetes.

Chemokines are also involved in the pathophysiology of obesity. Chronic inflammation is present in obesity due to increased mitochondrial metabolism and biogenesis, which results in the production of reactive oxygen species. CCL2, a member of the CC chemokine family [42], is a key factor in obesity-induced inflammation, as it attracts CCR2-expressing monocytes and favors their differentiation into M1 macrophages, which promote adipogenesis and increase the accumulation of adipose tissue [43,44]. Additionally, adipose tissue macrophage accumulation contributes to the development of insulin resistance [45]. Adipocytes release hormones, peptides, adipokines, and cytokines that can sensitize neurons and trigger hypersensitivity. Conversely, neurons release neuropeptides to communicate with inflammatory cells, which increases immune influx and polarization, thereby increasing inflammation and nociceptor sensitivity in white adipose tissue (WAT) [44]. These findings highlight the importance of the nervous and immune system crosstalk in regulating the pain response.

Chemokines are small (8 to 12 kD) molecules that were originally characterized by their ability to attract various immune cells, such as monocytes, lymphocytes, or neutrophils; thus, chemokines are considered to be pro-inflammatory. According to current knowledge, chemokines can be inflammatory or homeostatic [42]. Chemokines are divided into the following four groups based on the positioning of the N-terminal cysteine residues: C family, CC family, CXC family, and CX3C family. Chemokines have G-protein-coupled receptors through which they exercise multiple functions [42,46,47], such as angiogenic, antimicrobial [47], integrin activation, chemoattraction, leukocyte degranulation, and mediator release [46]. Several chemokines and chemokine receptors have also been found to be involved in nociception in neuropathic pain states [48,49,50,51,52,53,54], portraying them as potentially useful targets for pain management. Many members of the CC chemokine family are recognized as targets of successful pain therapy, as chemokine-neutralizing antibodies [49,52] and chemokine receptor antagonists [48,50,54,55,56] have been demonstrated to be effective at decreasing tactile and thermal hypersensitivity in constriction injury (CCI) [48,49,50,56,57] and/or streptozotocin (STZ)-induced diabetic [51,52,54,58] models of neuropathic pain. Among them, two chemokine receptors, namely CCR2 and CCR5, and their ligands deserve special attention, as their role in the development of hypersensitivity has been widely described in the literature [55,59,60], and their simultaneous modulation constitutes a new and satisfactory approach to relieving pain symptoms [48,54]. In addition, these treatments not only reduce pain symptoms but also improve opioid analgesic effects [49,50,51,55,58], which is noteworthy as chemokines decrease opioid receptor analgesic functions via cross-desensitization [61].

Chronic pain often results in low mood, stress, and even depression [62]. Stress and mood changes can cause metabolism impairment [63], including T1DM [64], T2DM [65], and obesity [66]. The effects of stress on glucose metabolism are mediated by a variety of hormones that are released in response to stress, which in turn results in elevated blood glucose levels, decreased insulin action, and obesity [63,64,65,66,67]. Musculoskeletal pain present in obese individuals is often the cause of reduced physical activity (PA), but at the same time, regular PA can improve health conditions by reducing inflammation and improving psychological outcomes like mood [68]. Interestingly, physical exercise can reduce the expression of pronociceptive CCL5 and its receptor, CCR5, in the adipose tissue of obese patients [69]. These data show that chemokines and their receptors deserve special attention as potential targets in chronic pain therapy, because pain management, and therefore also stress control, would be beneficial in improving the health status of obese and diabetic patients.

**Figure 1:**
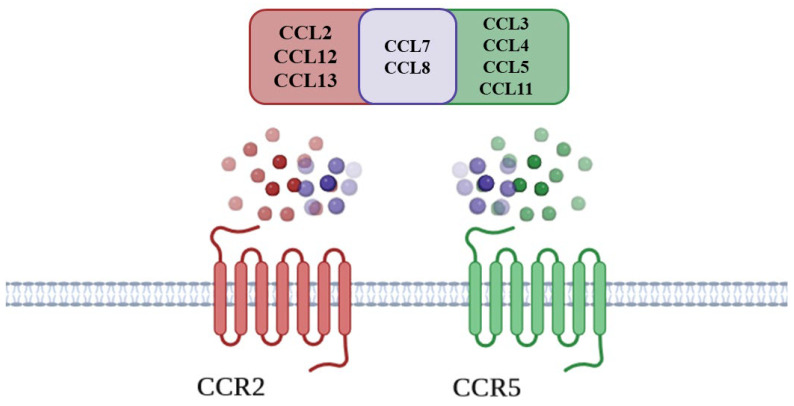
CCR2 and CCR5 and their endogenous ligands.

CCL7, CCL8, and CCL16 (which is a pseudogene in rodents [70]) are shared ligands of CCR2 and CCR5 [71,72]. CCR5 ligands also include CCL3, CCL4, CCL5, and CCL11 [71,72,73], whereas CCL2, CCL12, and CCL13 (not present in rodents) are the remaining ligands of CCR2 [71,72,74] (Figure 1).

The intrathecal injection of some CCR2 and CCR5 ligands, such as CCL2, CCL3, CCL5, CCL7, and CCL8, causes mechanical and thermal hypersensitivity in naïve mice. Additionally, upregulated mRNA and/or protein levels of these chemokines are present 2–28 days after CCI, suggesting that CCR2 and CCR5 ligands participate in the development and maintenance of neuropathic pain [57]. CCL2 [49], CCL3 [58], and CCL7 [49] decrease morphine-induced analgesia, and chemokine-neutralizing antibodies reverse the opioid antinociceptive effect. The activation of CCR2 and CCR5 decreases the chemotactic activities of μ- and δ-opioid receptors in vitro via heterologous desensitization [61]. Blockage of CCR2 and CCR5 by their antagonists, namely RS504393 [50], maraviroc [50,55], and cenicriviroc [48,50,54], has been demonstrated to alleviate pain in CCI and STZ models of neuropathic pain. These results suggest that CCR2 and CCR5, as well as their ligands, are important factors in regulating pain.

This review provides and discusses current knowledge of the involvement of CCR2, CCR5, and their ligands in diabetic neuropathy and obesity-induced hypersensitivity (summarized in Table 1 and Table 2), emphasizing evidence from rodent-based studies.

## 2. CCR2

In mouse models of diabetic neuropathy, CCR2 spinal mRNA levels are not changed in female mice but they are reduced in male diabetic mice 7 days after STZ induction. Additionally, CCR2 exhibits no statistically significant differences in protein levels in male and female diabetic mice compared to control mice [54]. Two weeks after STZ administration, when mechanical hypersensitivity develops, the CCR2 protein level is not changed in the T10 spinal cord of male diabetic rats [76]. Another study performed on diabetic rats has revealed similar results, as the CCR2 mRNA level is not changed in the spinal cord tissue of diabetic rats with mechanical or thermal hyperalgesia 3–4 weeks after STZ injection. Additionally, the CCR2 level is not affected by the RAP-103 multi-chemokine receptor (CCR2/CCR5/CCR8) antagonist [75]. In contrast, CCR2 mRNA and immunoreactivity levels are increased in the dorsal horn of the spinal cord of monkeys with T2DM [109]. Monocytes isolated from blood samples of T2DM patients express more CCR2 molecules per cell than control ones and, additionally, poor glycemic control increases CCR2 expression [77]. A meta-analysis of the diabetic patient gene expression profiles has demonstrated that CCR2 may be a promising biomarker for diabetes mellitus and diabetic peripheral neuropathy [40], but more research is needed on this topic.

At the age of 15 weeks, both diet-induced and genetically obese (*ob*/*ob*) mice present increased CCR2 mRNA levels in white adipose tissue compared with control mice [90]. Compared with that in control mice, CCR2 expression in epididymal adipose tissue from diet-induced obese mice fed a high-fat diet for 24 weeks is elevated, and treatment with pioglitazone, which is used to treat diabetes, results in lower CCR2 expression in comparison to obese nontreated mice. Additionally, *Ccr2* knockout mice show decreased food intake, as measured over a 6-week period, and lower fasting glucose and insulin concentrations, as measured after 20 weeks of high-fat diet consumption [91]. Obese mice with the *Ccr2^−/−^* genotype show increased insulin sensitivity and greater glucose tolerance [91], which additionally indicates that CCR2 plays a role in regulating glucose levels in obesity. A study performed on obese children has revealed that CCR2+ monocyte subsets are increased in blood samples from boys but not from girls [92]. CCR2 is important in obesity, as CCR2-expressing monocytes are recruited to white adipose tissue, which leads to cytokine production and stimulation of adipogenesis, contributing to WAT deposition [44]. However, no data on the role of CCR2 in obesity-induced hypersensitivity have been reported, and exploratory studies in animal models are needed to address this topic.

## 3. CCR5

In a diabetic neuropathy model, CCR5 mRNA levels are increased in the lumbar region of the spinal cord in female diabetic mice, but there is no statistically significant difference between male diabetic and control mice on day 7 after STZ injection [54]. Additionally, CCR5 protein levels are decreased in male, but not female, diabetic mice [54]. Three to four weeks after STZ injection, when diabetic neuropathic pain is developed, CCR5 mRNA levels do not differ between diabetic and control rats in the same region of the spinal cord. Moreover, the CCR5 level is decreased by the RAP-103 multi-chemokine receptor antagonist, which also reduces mechanical and thermal hypersensitivity [75]. In contrast, T2DM monkeys present increased CCR5 mRNA levels in the dorsal part of the spinal cord horn, compared with control monkeys [109]. Monocytes and lymphocytes isolated from patients with T2DM with a 5-year or shorter course (SDM group) and patients with a course of more than 5 years (LDM group) display increased surface CCR5 lymphoid ratios in both groups and increased CCR5 mononuclear ratios in the LDM group, compared with those in the SDM group and the control group [78].

CCR5 mRNA and protein levels are increased in the WAT of both genetically (*ob*/*ob*)- and diet-induced obese mice at 15 and 20 weeks of age [90]. At week 20 of age, mice with the *Ccr5^−/−^* genotype do not show differences in food intake, body weight, adipose tissue weight, or fasting plasma glucose levels, when fed both normal and high-fat diets compared to wild-type mice. However, *Ccr5^−/−^* mice fed only a high-fat diet exhibit 50% lower insulin levels and greater insulin sensitivity compared to wild-type mice. Additionally, plasma concentrations of free fatty acids and triglycerides are lower in *Ccr5* knockout mice than in wild-type mice. In *Ccr5^−/−^* mice, diet-induced glucose intolerance and hyperinsulinemia are improved compared to those in wild-type mice fed the same diet [90]. Although CCR5 may be important in regulating insulin sensitivity, its level does not change in rodent models of STZ-induced diabetic neuropathy; however, RAP-103 treatment decreases the mRNA level of CCR5 and alleviates tactile and thermal hypersensitivity, suggesting that CCR5 participates in nociceptive transmission in the chronic pain state. Thus, further research is needed to assess the precise role of CCR5 in these conditions, including diabetic neuropathy and obesity-induced hypersensitivity.

## 4. CCL2

CCL2 is a widely researched chemokine, and many studies regarding this factor are available; however, the role of CCL2 in chronic pain is not fully understood. A model of STZ-induced diabetic neuropathy has revealed increased CCL2 mRNA levels in the lumbar region of the spinal cord in male mice on day 7 after STZ injection [51], which was confirmed by our recent study. However, CCL2 protein levels are not changed in either male or female diabetic mice, as measured only on day 7 after STZ treatment [54]. In rats with diabetic neuropathy, the CCL2 expression level is increased 3–4 weeks after STZ injection in sciatic nerve tissue but not in the spinal cord [75]. Four weeks after STZ injection, CCL2 protein levels are increased in the spinal cord and in the sciatic nerve tissue of diabetic rats with developed mechanical and thermal hypersensitivity [80]. In addition, research on New Zealand obese diabetic mice (NZO/HILtJ) has indicated that the CCL2 mRNA level is elevated at the spinal cord level [79]. CCL2 mRNA levels are increased in the WAT of genetic and high-fat diet-induced obesity and genetic diabetes in mice [94]. Typically, in mouse models of obesity, many studies have shown increased CCL2 levels in adipocytes and/or plasma [90,93], but no data on the role of CCL2 in obesity-induced hypersensitivity have been reported. Several studies performed in humans have indicated that CCL2 levels are increased in both diabetes [77,81,82] and obesity [95,96]. In monocytes, derived from the blood of T1DM patients, *Ccl2* levels are increased [82]. CCL2 protein levels are also elevated in the serum of T2DM patients, and, interestingly, this level is correlated with markers of insulin resistance, such as BMI [77]. In contrast, in blood samples from T1DM patients, the CCL2 protein level is lower than that in control samples, but it is elevated in patients with diabetes complications. CCL2 levels increase with disease duration and patient age, but no significant difference is present between sexes [81]. A similar result has been obtained in an STZ-induced mouse model of diabetes, with no statistically significant differences in CCL2 protein levels between male and female diabetic mice [54]. Research has shown that CCL2 mRNA levels in the serum and biopsies of subcutaneous abdominal adipose tissue are increased in obese patients compared with lean patients [96]. In adipocytes isolated from obese patients, CCL2 mRNA levels correlate with BMI and circulating CCL2. Additionally, weight loss causes a 20% decrease in circulating CCL2 [95], which suggests that this chemokine plays a role in obesity-related complications.

## 5. CCL3

CCL3 is involved in diabetic neuropathy as a neutralizing antibody against this chemokine reduces STZ-induced tactile and thermal hypersensitivity measured 7 days after STZ injection. Additionally, the CCL3 protein level is increased at the spinal cord level in diabetic male mice compared with control mice [58]. In contrast, the CCL3 mRNA level is not significantly different between STZ-induced diabetic and control male and female mice [54]. Rats with diabetic neuropathy have increased *Ccl3* levels in the spinal cord, but not in the sciatic nerve, 3–4 weeks after STZ injection [75]. Another study in rats has revealed that 4 weeks after STZ induction, when tactile and thermal sensitivity is developed, the CCL3 protein levels in the spinal cord and sciatic nerve increase in male diabetic rats [80]. Moreover, research conducted in diabetic monkeys has shown that the CCL3 mRNA level is increased in the dorsal horn of the spinal cord [109]. To date, only one study has considered the role of CCL3 in human diabetes. A study conducted on gestational diabetes mellitus patients has revealed that there is no difference in the CCL3 expression level in amniotic membrane-resident macrophages between pregnant women with gestational diabetes and controls [83]. Research on animals has suggested that CCL3 is involved in the neuropathic pain caused by diabetes, suggesting that it may be a prominent target for pain treatment. Further, increased CCL3 levels have also been observed in obese individuals. CCL3 mRNA levels in white adipose tissue are increased in both genetically (*ob*/*ob*)- and diet-induced obese mice at 15 and 20 weeks of age [90]. Compared with control mice, diet-induced obese male and female mice present increased CCL3 expression levels in white adipose tissue at week 24 of age [97]. An increase in CCL3 levels in serum or adipose tissue is associated with the presence of metabolic syndrome [98]. Circulating samples from obese girls, but not boys, present elevated CCL3 protein levels, and this level is correlated with BMI [99]. Thus, CCL3 may constitute a common target for effective pharmacotherapy in obese individuals, who suffer from other ailments, such as diabetes and hypersensitivity.

## 6. CCL4

In a diabetic neuropathy model, the CCL4 protein level is not changed at the spinal cord level in male mice compared with control mice 7 days after STZ injection [58], which is consistent with our recent finding that at the same time point CCL4 mRNA level is not changed in STZ-induced male or female mice [54]. Nevertheless, single intrathecal administration of CCL4 increases mechanical and thermal hypersensitivity in diabetic mice, which highlights the role of CCL4 in nociception [58]. Clinically, the role of CCL4 in diabetes has not yet been well established; however, study on diabetic patients with and without diabetic retinopathy has shown that in retinal glial cells, the CCL4 protein level is lower in diabetic patients without diabetic retinopathy than in controls [84]. Placental tissues collected from female patients with gestational diabetes mellitus also exhibit lower CCL4 protein levels than those from patients with normal glucose tolerance [85]. In contrast, genetically (*ob*/*ob*)- and diet-induced obese mice have increased CCL4 mRNA levels in white adipose tissue at weeks 15 and 20 of age [90]. Another study has revealed that diet-induced obese male and female mice present increased CCL4 expression levels in white adipose tissue at week 24 of age compared with control mice fed a normal chow diet [97]. Circulating CCL4 protein levels are elevated in obese patients compared with lean patients; moreover, this level decreases 10 months after bariatric surgery [100]. CCL4 protein levels are increased in circulating samples from obese girls, but not obese boys, and this level corresponds with the BMI [99]. These data suggest that CCL4 may not be crucial in the development of diabetic neuropathy; however, elevated CCL4 is present in obese individuals. Thus, further research is needed to determine whether CCL4 is connected to hypersensitivity related to obesity.

## 7. CCL5

The role of CCL5 in diabetic neuropathy has recently been investigated in an STZ-induced mouse model of diabetes. On day 7, when tactile and thermal hypersensitivity is developed, and the CCL5 mRNA, but not protein, level is increased in the lumbar part of the spinal cord in both male and female mice [54]. A meta-analysis has revealed that the concentration of circulating CCL5 is greater in T1DM patients than in controls; however, there was a high level of heterogeneity among included studies (*I*^2^ = 99%) [86]. Research has demonstrated that there is no difference in CCL5 protein levels in retinal glial cells between diabetic patients and the controls [84]. An Iranian study on more than 100 T1DM patients has revealed that the CCL5 circulating protein level is increased in diabetic patients and that this level rises with the duration of the disease, but there is no significant difference between male and female patients. Additionally, CCL5 levels are increased in patients suffering from diabetes complications [81]. Compared with the controls, CCL5 plasma mRNA levels are significantly lower in patients with shorter (5 years or less) and longer (more than 5 years) durations of T2DM, and there is no difference between the diabetic groups. Furthermore, there is no significant difference in CCL5 levels between sexes [78]. CCL5 probably also plays an important role in obesity as shown in animal models. At 15 and 20 weeks of age, both genetically (*ob*/*ob*)- and diet-induced obese mice have increased CCL5 mRNA levels in white adipose tissue [90]. Compared with control mice fed a normal diet, diet-induced obese male and female mice, at week 24 of age, present elevated CCL5 expression levels in white adipose tissue [97]. CCL5 expression in serum and white adipose tissue is increased in obese women compared with lean women [101]. These data suggest that CCL5 may be important in type 1 diabetes and obesity, but more research is needed to evaluate the role of CCL5 in diabetic neuropathy and obesity-induced hypersensitivity.

## 8. CCL7

CCL7 mRNA, but not protein, levels are increased in the lumbar region of the spinal cord in both male and female mice on day 7 after STZ administration, when mechanical and thermal hypersensitivity develop [54]. Compared with those from control subjects, CD14-positive monocytes, isolated from patients’ blood, present increased CCL7 mRNA levels in all groups of diabetic subjects (those with juvenile-onset type 1 diabetes, adult-onset type 1 diabetes, latent autoimmune diabetes, and T2DM) [82]. In contrast, another study has demonstrated that the circulating CCL7 concentration is decreased in T1DM patients [87]. However, limited data suggest that further research is needed to fully understand whether and how CCL7 participates in the development of diabetic neuropathy. In turn, compared with normal diet-fed mice, high-fat diet-fed mice present increased CCL7 mRNA expression levels in adipose tissue. Moreover, mice fed on a high-fat diet have significantly lower CCL7 mRNA levels, when administered with naringenin, a flavonoid present in citrus fruits, which has proven pharmacological effects, for example anti-diabetic and anti-inflammatory [102]. Compared with that in control lean mice, CCL7 expression is elevated in the epididymal adipose tissue of obese and insulin-resistant mice, maintained on a high-fat diet for 24 weeks. Additionally, feeding mice a high-fat diet for 22 weeks, followed by an additional 2 weeks of the same diet supplemented with pioglitazone, an antidiabetic medication, results in lower CCL7 expression [91]. Compared with control mice, both genetic (*ob*/*ob*)- and diet-induced obese mice, at the age of 15 weeks, present increased CCL7 mRNA levels in white adipose tissue [90]. *Ccl7* levels are increased in adipose tissue from diabetic obese patients, but not in diabetic lean or overweight subjects [88]. Additionally, CCL7 expression is upregulated in the omental and subcutaneous adipose tissue of obese patients, compared with lean controls [103]. As increased CCL7 levels are found in diabetes and obesity, preliminary studies on rodent models of hypersensitivity present in obesity are needed to assess the significance of CCL7 in these conditions.

## 9. CCL8

Our recent study has revealed that the CCL8 mRNA level is elevated at the spinal cord level in female, but not in male, diabetic mice 7 days after STZ injection when tactile and thermal hypersensitivity develop. However, there were no differences in CCL8 protein levels in both male and female diabetic mice [54]. Compared with those in control patients, CCL8 protein levels are increased in the retinal glial cells of diabetic patients [84]. Compared with those from overweight and control patients, ex vivo TCR-induced leukocytes, isolated from the blood of diabetic and overweight children, display significantly greater CCL8 expression in diabetic patients. In leukocytes of overweight children, CCL8 expression is unchanged [89]. CCL8 expression is increased in the epididymal adipose tissue of obese and insulin-resistant mice fed a high-fat diet for 24 weeks; however, treatment with pioglitazone does not affect the CCL8 level [91]. Similarly, the CCL8 mRNA level is also increased at 15 and 20 weeks of age in white adipose tissue of both genetically (*ob*/*ob*)- and diet-induced obese mice [90]. Although, abundant research on the role of CCL8 in diabetes and obesity is lacking, existing data suggest that CCL8 has a role in diabetes, diabetic neuropathy, and the progression to obesity. Thus, CCL8 is a promising molecule to examine in related topics, such as obesity-induced hypersensitivity, and identifying new disease markers and new drugs for treating chronic pain. Additionally, our recent study has revealed for the first time differences in CCL8 levels between sexes, which is particularly important due to the disparity in experiencing pain in chronic conditions between men and women. Thus, our findings may provide insight into the mechanism underlying these differences, allowing more individualized treatments to be developed and used for chronically ill patients.

## 10. CCL11

Compared with that in control mice, CCL11 mRNA levels are not changed at the spinal cord level in both male and female STZ-induced diabetic neuropathy model mice on day 7 [54]. Studies on diabetic patients have shown that the circulating CCL11 protein level is increased in T1DM patients and that this level is correlated with the duration of the disease and the age of the patient, but there are no differences between the sexes [81]. CCL11 mRNA concentrations are decreased in blood cord samples from women with gestational diabetes mellitus [85]. In contrast, compared with lean control mice fed a normal diet, obese male and female mice, fed a high-fat diet for 26 weeks, present significantly increased CCL11 mRNA levels in adipose tissue and elevated CCL11 protein levels in blood samples. CCL11 mRNA levels in adipose tissue are positively correlated with serum protein levels. Additionally, compared with explants from lean individuals, adipose tissue explants from obese mice, cultured ex vivo for 24 h, present increased CCL11 secretion levels. Compared with control lean subjects, obese patients with metabolic syndrome show increased serum CCL11 levels, and these levels decrease after weight loss [104]. In contrast, another study has demonstrated that the level of circulating CCL11 protein is lower in obese patients than in lean patients [105]. Available data suggest that CCL11 may be important in later phases, rather than in the beginning, of diabetic neuropathy. Although CCL11 has been demonstrated to be increased in patients with diabetes and obesity, further research is needed to establish the role of CCL11 in diabetic neuropathy and obesity-induced hypersensitivity.

## 11. CCL12

In the mice model of neuropathic pain, caused by chronic constriction injury of the sciatic nerve, spinal CCL12 mRNA level is increased on days 2 and 7 in male mice, but it was proven not to have pro-nociceptive nor analgesic properties [49]. In the STZ-induced mouse model of diabetic neuropathic pain on day 7, the CCL12 spinal mRNA level is increased in female mice, but there are no significant differences between diabetic and control male mice [54]. CCL12 has not been investigated in patients with diabetes and obesity, nor in mouse models of obesity and obesity-induced hypersensitivity. Current data suggest that CCL12 may not be important in nociception in chronic pain, such as diabetic neuropathy and obesity-induced hypersensitivity. Thus, more studies are needed to fully understand the role of CCL12 in these states and the differences present between the sexes.

## 12. CCL13

CCL13 is absent in rodents [110], thus, it is poorly characterized in the literature [110]. However, many studies have suggested that CCL13 is involved in many chronic inflammatory diseases, as a key molecule of immune cell recruitment [111,112]. Although there is no information available about the function of this chemokine in chronic pain, data suggesting its involvement in abdominal pain in irritable bowel syndrome [113] and osteoarthritis [114] warrant further studies to determine its role in this context. CCL13 has been detected in the serum of obese patients, and its increased levels are correlated with parameters related to metabolic syndrome, such as insulin resistance, body mass index, and waist circumference. Other studies have also shown that elevated CCL13 levels are associated with BMI [106,108], and adipocytes from obese postmenopausal women show higher CCL13 expression than those from lean ones [107]. These findings indicate that CCL13 may be a biomarker of chronic inflammation in obesity, and the lack of literature regarding this chemokine’s role in the development of chronic pain and hypersensitivity highlights a need for further research in this area.

## 13. Future Prospects

**Figure 2:**
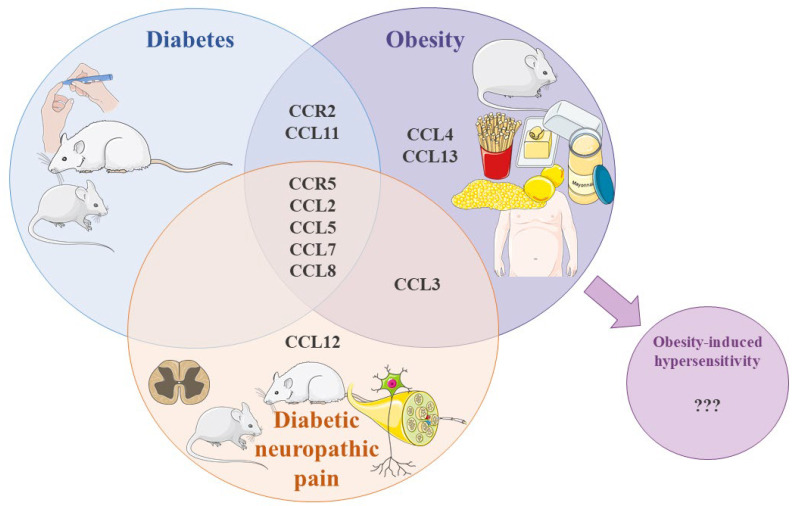
Involvement of CCR2, CCR5, and their ligands in diabetes, diabetic neuropathic pain, obesity, and obesity-induced hypersensitivity.

Neuroinflammation, including the release of inflammatory mediators, such as chemokines, in the peripheral and central nervous systems, plays an important role in the initiation and maintenance of chronic pain [115]. Chemokines, including CCR2 and CCR5 ligands, have been demonstrated to have pronociceptive properties, and their levels are increased in CCI and STZ models of neuropathic pain [48,49,50,51,52,53,54]. Research has shown that elevated CCL2 [77,82,90,93,94,95,96], CCL5 [81,86,90,97,101], CCL7 [82,88,90,91,102,103], CCL8 [54,84,89,90,91], CCL11 [81,104], CCR2 [40,77,90,91,92], and CCR5 [54,78,90] levels can be found in the samples (circulating, adipose tissue or others) from subjects (rodents and/or humans) with diabetes and obesity; additionally, CCL3 [90,97,98,99], CCL4 [90,97,99,100], and CCL13 [106,107,108] levels are increased in subjects with obesity (Figure 2), but some results are contradictory or exhibit sex differences (Table 1 and Table 2).

Importantly, in circulating samples from diabetic and/or obese patients, increased CCR2 [40], CCL2 [77,95], CCL3 [98,99], CCL4 [99,100], CCL5 [81,86,101], CCL11 [81,104], and CCL13 [106,108] mRNA and/or protein levels can be found, with some chemokines exhibiting sex-related differences; for example, increased levels of CCL3 and CCL4 in obese patients were observed in women but not men [99]. Taking the above into account, differences in the expression of the presented chemokines are not only dependent on the etiology of neuropathy but also on the sex. A review and comparison of these variables can be used to create a coherent pattern of biomarkers determined by the etiology of pain and sex, which would be of fundamental importance in future diagnostics. Moreover, it would be especially valuable to inspect chemokine levels in blood samples from patients suffering from diabetic neuropathy to possibly identify new biomarkers of the disease. It would be particularly important as available screening methods are limited and semi-quantitative [18,116]. Current tests evaluating the functioning of nerve fibers include the temperature perception test and the pinprick pain perception test; meanwhile, long nerve fiber function is assessed by the vibration perception test, the monofilament touch perception test, and the evaluation of ankle reflexes, but the problem that those tests face is that 50% of patients with diabetic neuropathy are asymptomatic. Confirmation of diabetic neuropathy diagnosis is complex and rarely performed [116], and can be achieved through electrodiagnosis, which requires special personnel and equipment [117]. The need for quantitative and easily accessible tests from samples that are simply acquirable remains unfulfilled and future studies on diabetic neuropathy should focus on making this idea possible.

**Figure 3:**
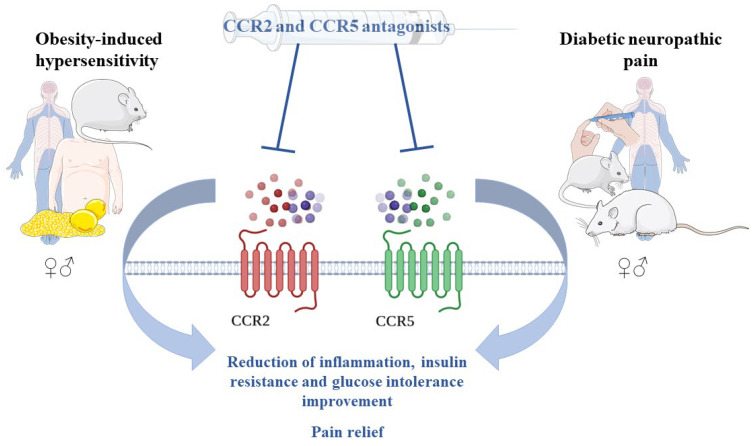
Proposed treatment option and outcomes for diabetic neuropathic pain and obesity-induced hypersensitivity.

Additionally, it has been shown that CCR5 [54], CCL2 [51,54,75,79,80], CCL3 [58,75,80], CCL5 [54], CCL7 [54], CCL8 [54], and CCL12 [54] spinal mRNA and/or protein levels are elevated in rodent models of diabetic neuropathy (Figure 2), with some displaying sex-related diversity. For example, CCL2 mRNA and protein levels are increased in males [51,54,75,80], while in females it exhibits no differences [54]. CCL5 and CCL7 protein levels are decreased in males, whereas in females no changes were observed. Moreover, elevated expression levels of CCL8 and CCL12 were demonstrated in females, but not in males [54]. However, these chemokines and their receptors have not been studied in a model of obesity-induced hypersensitivity, which highlights a new pathway for future research in terms of discovering new potential targets for pain therapy in this context. Chemokine-neutralizing antibodies [49,52] and chemokine receptor antagonists have been demonstrated to be successful in alleviating pain in neuropathies of different etiologies, including diabetes [48,50,51,54,55,56,58]. Further, maraviroc, a CCR5 antagonist, improves insulin resistance and attenuates pancreatic β-cell dysfunction in mice fed a high-fat diet [118]. A recent study of diabetic neuropathy has reported that long-term administration of cenicriviroc, a dual CCR2/CCR5 antagonist, results in better pain relief compared to morphine alone or the combination of cenicriviroc and morphine, suggesting that blocking CCR2 and CCR5 simultaneously may be a better and safer treatment option than the administration of strong analgesics, such as opioids. Interestingly, cenicriviroc’s strong analgesic effect was present in both male and female mice [54], which is an especially important result as women suffering from diabetic neuropathy develop painful symptoms more often than men [5,12,13]. Moreover, treatment with PF4178903, another dual CCR2/CCR5 antagonist, reduces adipose tissue macrophage infiltration, lowers serum proinflammatory cytokines levels, and improves insulin resistance and glucose intolerance in mice fed a high-fat diet [119]. These findings demonstrate that dual CCR2/CCR5 antagonists may reduce chronic inflammation more effectively, improve opioid tolerance, and reduce pain symptoms (Figure 3). Additionally, treating painful symptoms improves mood and reduces stress [62], which adds an additional advantage in improving health conditions in diseases with impaired metabolism like diabetes and obesity. In summary, the collected data of CCR2, CCR5, and their ligand level changes are expected to help identify a biological marker of susceptibility to chronic pain in both diabetic and obese individuals, which will allow the identification of new targets for effective painkillers that could be used in these specific painful conditions. Further research on this topic would be especially valuable for clinicians in need of new, better screening methods and personalized pain therapy, which takes into account pain etiology and sex differences. Available data suggest that CCR2, CCR5, and their ligands might be great targets to start with to overcome the stagnation in the ineffectiveness of chronic pain treatment.

## Figures and Tables

**Table 1:** Changes in the mRNA and protein levels of CCR2, CCR5, and their ligands in diabetic neuropathic pain rodent models and diabetic patients. Abbreviations: **↑** increase; **↓** decrease; ─ no change; **↑↓**─ male; **↑↓**─ female; **↑↓**─ both; **↑↓**─ not distinguished; ND no data available; * diabetic obese subjects.

Receptor/ Chemokine	Diabetic Neuropathic Pain Rodent Models		Diabetic Patients			
	Spinal Cord		Circulating		Others	
	mRNA	Protein	mRNA	Protein	mRNA	Protein
CCR2	**↓**[54] **─**[54]	**─**[54] **─**[75,76]	**↑**[40]	ND	**↑**[40]	**↑**[77]
CCR5	**─**[54,75] **↑**[54]	**─**[54]	ND	ND	ND	**↑**[78]
CCL2	**↑**[51,54,75] **─**[54] **↑**[79]	**↑**[80] **─**[54]	ND	**↑**[77] **↓**[81]	**↑**[82]	ND
CCL3	**─**[54] **↑**[75]	**↑**[58,80]	ND	ND	**─**[83]	ND
CCL4	**─**[54]	**─**[58]	ND	ND	ND	**↓**[84] **↓**[85]
CCL5	**↑**[54]	**↓**[54] **─**[54]	**─**[78]	**↑**[81] **↑**[86]	ND	**─**[84]
CCL7	**↑**[54]	**↓**[54] **─**[54]	ND	**↓**[87]	**↑**[82] **↑**[88] *	ND
CCL8	**─**[54] **↑**[54]	**─**[54]	ND	ND	**↑**[89]	**↑**[84]
CCL11	**─**[54]	ND	**↓**[85]	**↑**[81]	ND	ND
CCL12	**─**[54] **↑**[54]	ND	ND	ND	ND	ND
CCL13	ND	ND	ND	ND	ND	ND

**Table 2:** Changes in the mRNA and protein levels of CCR2, CCR5, and their ligands rodent models of obesity and obese patients. Abbreviations: **↑** increase; **↓** decrease; ─ no change; **↑↓**─ male; **↑↓**─ female; **↑↓**─ both; **↑↓**─ not distinguished; ND no data available; * diabetic obese subjects.

Receptor/ Chemokine	Rodent Models of Obesity		Obese Patients					
	Adipose Tissue		Adipose Tissue		Circulating		Other	
	mRNA	Protein	mRNA	Protein	mRNA	Protein	mRNA	Protein
CCR2	**↑**[90,91]	ND	ND	ND	ND	ND	ND	**↑**[92]
CCR5	**↑**[90]	**↑**[90]	ND	ND	ND	ND	ND	ND
CCL2	**↑**[90,93,94]	ND	**↑**[95] **↑**[96]	ND	**↑**[95]	ND	ND	ND
CCL3	**↑**[90,97] **↑**[97]	ND	**↑**[98]	ND	**↑**[98]	**─**[99] **↑**[99]	ND	ND
CCL4	**↑**[90,97] **↑**[97]	ND	ND	ND	ND	**─**[99] **↑**[99] **↑**[100]	ND	ND
CCL5	**↑**[90,97] **↑**[97]	ND	**↑**[101]	ND	**↑**[101]	ND	ND	ND
CCL7	**↑**[90,91,102]	ND	**↑**[103] **↑**[88] *	ND	ND	ND	ND	ND
CCL8	**↑**[90,91]	ND	ND	ND	ND	ND	**─**[89]	ND
CCL11	**↑**[104]	ND	ND	ND	ND	**↑**[104] **↓**[105]	ND	ND
CCL12	ND	ND	ND	ND	ND	ND	ND	ND
CCL13	ND	ND	**↑**[106] **↑**[107]	ND	ND	**↑**[106,108]	ND	ND
